# Clinically Actionable Prevention and Management of Major Complications During Durable Left Ventricular Assist Device Support: A Contemporary Narrative Review

**DOI:** 10.14740/cr2283

**Published:** 2026-10-24

**Authors:** Wei Yong Sheng, Tian Sheng Tang, You Shi Wu, Hussain Asif, Saifane Zakaria, Da Fa Zhang

**Affiliations:** a Department of Cardiovascular Surgery, The First Affiliated Hospital of Wannan Medical University (Yijishan Hospital), Wuhu, Anhui, China

**Keywords:** Durable left ventricular assist device, Advanced heart failure, Right heart failure, Hemocompatibility, Bleeding, Multidisciplinary follow-up

## Abstract

Durable left ventricular assist devices (LVADs) have become an established therapy for selected patients with advanced heart failure as bridge-to-transplant or destination therapy. Despite major progress in continuous-flow pump design and perioperative care, long-term benefit remains constrained by a recurrent burden of right heart failure, aortic regurgitation, pump thrombosis, stroke, bleeding, infection, and end-organ dysfunction. Because these events often arise from the interaction of altered hemodynamics, hemocompatibility-related injury, chronic antithrombotic exposure, and the practical complexity of longitudinal device care, a complication-centered management framework is clinically useful. This contemporary narrative review focuses on actionable prevention, surveillance, and treatment strategies for the major complications encountered during durable LVAD support. Rather than providing a broad device-oriented overview, we emphasize clinically relevant decision points across the perioperative and long-term course, including right ventricular protection, antithrombotic balance, structured monitoring for aortic regurgitation and hemolysis, prevention of driveline infection, and multidisciplinary outpatient follow-up. By organizing current evidence around practical management pathways, this review aims to support earlier recognition of adverse events and more durable long-term support.

## Introduction

Durable left ventricular assist devices (LVADs) are now an established form of mechanical circulatory support for selected patients with advanced heart failure, both as bridge-to-transplant and as destination therapy. Contemporary continuous-flow systems, particularly fully magnetically levitated centrifugal pumps, have improved device durability, hemocompatibility, survival, and quality of life compared with earlier-generation axial-flow pumps [1–3]. Design changes such as contact-free rotor suspension and wider blood-flow gaps are intended to reduce blood trauma, although clinical outcomes and management requirements remain device-specific [2, 3]. In our center, the practical experience informing this review is based primarily on HeartMate 3 and CorHeart 6 durable LVAD programs; therefore, the recommendations below are written for contemporary continuous-flow devices rather than for older pulsatile or axial-flow platforms. Device-specific trial findings should not be extrapolated between HeartMate 3 and CorHeart 6 without supporting evidence.

However, the major determinant of long-term success is no longer implantation alone. Instead, outcomes are increasingly shaped by the prevention, early recognition, and longitudinal management of major adverse events. Right heart failure (RHF), aortic regurgitation, pump thrombosis, stroke, bleeding, infection, and progressive end-organ dysfunction remain leading causes of rehospitalization, functional decline, and late mortality during LVAD support [2–5]. These complications rarely reflect isolated technical failures. More often, they emerge from the interaction of altered ventricular loading, continuous-flow physiology, hemocompatibility-related injury, chronic antithrombotic exposure, patient-device mismatch, and the operational demands of prolonged outpatient support.

An important challenge in the current literature is that complication domains are often discussed separately, whereas daily LVAD care requires integrated decisions across hemodynamics, anticoagulation, infection prevention, imaging surveillance, and multidisciplinary follow-up. For this reason, a clinically actionable synthesis should not simply catalogue complications; it should help clinicians recognize how these events evolve across the perioperative and chronic phases of support, where surveillance is most valuable, and when management thresholds should change.

This contemporary narrative review therefore focuses on the practical prevention, surveillance, and management of the major complications encountered during durable LVAD support. We specifically emphasize perioperative right ventricular (RV) protection, hemocompatibility-related adverse events, aortic valve surveillance, driveline infection prevention, end-organ monitoring, and multidisciplinary long-term care. Our aim is to provide a management-oriented framework that helps translate current evidence into day-to-day clinical practice rather than to present an exhaustive systematic review of all LVAD-related literature [5]. To make this framework operational, we summarize key complication domains, surveillance signals, and escalation thresholds in Table 1, provide a phase-based follow-up checklist in Table 2, and outline an integrated four-domain surveillance model in Figure 1. These tables are intended as a practical synthesis of contemporary evidence and expert clinical practice, rather than as validated risk algorithms or prescriptive institutional protocols.

**Table 1:** Practical Framework for Prevention, Surveillance, and Escalation of Major Complications During Durable LVAD Support

Complication domain	Main mechanisms	Surveillance signals	Prevention focus	Escalation triggers
Right heart failure	RV preload-afterload mismatch, septal shift, pulmonary hypertension, tricuspid regurgitation	CVP or right atrial pressure, PAP/PVR, PAPi, RA/PCWP ratio, TAPSE, RV S', FAC or strain when available, TR severity, septal position, congestion, renal/hepatic indices, inotrope need	Preoperative decongestion, pulmonary vascular optimization, rhythm and ventilatory optimization, intraoperative TEE-guided pump-speed titration, planned RV support strategy in high-risk patients	Persistent low output, escalating inotropes or vasopressors, refractory venous congestion, worsening renal/hepatic function, lactate rise, need for temporary RV mechanical support
Aortic regurgitation	Reduced aortic valve opening, commissural fusion, altered aortic root flow, low pulsatility, aortic root remodeling	Symptoms, pulse pressure/MAP, aortic valve opening frequency, AR severity on echocardiography, LV size, filling pressures, pump flow/power trends	Blood pressure control, speed optimization to avoid persistent valve closure when feasible, serial valve assessment, consideration of concomitant valve intervention at implant when indicated	Hemodynamically significant recirculation, recurrent HF symptoms, repeated hospitalization, progressive LV dilation, consideration of TAVR, redo SAVR, valve repair/replacement or closure
Pump thrombosis/thromboembolism	Flow disturbance, blood-material interaction, subtherapeutic anticoagulation, infection, cannula/graft malposition, low-flow states	LDH, plasma-free hemoglobin, hemoglobinuria/dark urine, pump power or flow change, alarms, neurologic events, echo ramp response, CT for cannula/outflow graft assessment	Stable VKA-based anticoagulation, protocolized perioperative heparin transition, infection control, BP control, device-position assessment, adherence support	Rising hemolysis markers with device dysfunction, recurrent low-flow/power abnormalities, stroke/systemic embolism, refractory hemolysis, need for thrombolysis or pump exchange
Bleeding	Shear-related platelet dysfunction, acquired von Willebrand syndrome, angiodysplasia, antithrombotic exposure	Hemoglobin trend, occult/overt GI bleeding, transfusion need, INR stability, antiplatelet exposure, renal/liver dysfunction	Individualized antithrombotic intensity, anemia surveillance, BP and device optimization, avoidance of unnecessary antiplatelet therapy in selected HM3 recipients	Recurrent bleeding, hemodynamic instability, repeated anticoagulation interruption, transfusion dependence, need for endoscopy or multidisciplinary antithrombotic revision
Driveline/device infection	Percutaneous exit-site vulnerability, trauma, dressing disruption, diabetes, malnutrition, prolonged support duration	Exit-site pain, erythema diameter, warmth, induration, drainage amount/character, granulation tissue, driveline instability, DESTINE or modified Sharp Memorial score, cultures, CRP/WBC, blood cultures, ultrasound or CT/PET-CT when deep infection suspected	Driveline stabilization, standardized dressing protocol, exit-site hygiene, patient/caregiver education, early treatment of trauma or drainage, outpatient surveillance	Deep/tunnel infection, pump-pocket or pump infection, bacteremia, recurrent infection, systemic illness, transplant/device-exchange implications
Renal and end-organ dysfunction	Venous congestion, low output, perioperative injury, RHF, recurrent adverse events, nephrotoxic exposure	Creatinine/eGFR, urine output, bilirubin/transaminases, lactate, nutrition/frailty, diuretic response, congestion markers	Congestion control, RV protection, medication review, renal-dose adjustment, early complication treatment, nutritional and rehabilitation support	Progressive renal/hepatic dysfunction, refractory congestion, worsening frailty or malnutrition, transplant candidacy impact, need for higher-intensity follow-up

AR: aortic regurgitation; BP: blood pressure; CRP: C-reactive protein; CT: computed tomography; CVP: central venous pressure; eGFR: estimated glomerular filtration rate; FAC: fractional area change; GI: gastrointestinal; HF: heart failure; HM3: HeartMate 3; INR: international normalized ratio; LDH: lactate dehydrogenase; LV: left ventricular; LVAD: left ventricular assist device; MAP: mean arterial pressure; PAP: pulmonary artery pressure; PAPi: pulmonary artery pulsatility index; PET: positron emission tomography; PVR: pulmonary vascular resistance; RA/PCWP: right atrial/pulmonary capillary wedge pressure; RHF: right heart failure; RV: right ventricular; SAVR: surgical aortic valve replacement; TAPSE: tricuspid annular plane systolic excursion; TAVR, transcatheter aortic valve replacement; TEE: transesophageal echocardiography; TR: tricuspid regurgitation; VKA: vitamin K antagonist; WBC: white blood cell.

**Table 2:** Phase-Based Surveillance Checklist for Durable LVAD Recipients

Care phase	Core clinical questions	Minimum surveillance elements	Common action points
Preoperative/candidacy	Is the patient likely to tolerate LV unloading and long-term support?	RV function, TAPSE/RV S'/FAC or strain, TR severity, RA pressure, PAP/PVR, PCWP, PAPi, RA/PCWP ratio, renal/hepatic function, nutrition/frailty, infection risk, psychosocial support	Optimize congestion, treat reversible pulmonary hypertension, plan RV support strategy, address infection/nutrition, educate patient and caregivers
Early postoperative	Is effective forward flow achieved without RV failure, bleeding, or major hemocompatibility disturbance?	Hemodynamics, echo/TEE, septal position, LV size, AV opening, volume status, chest tube output, coagulation profile, renal/liver tests, lactate, urine output, LDH/plasma-free hemoglobin, device trends	Adjust pump speed, titrate inotropes/vasodilators, restart heparin only after bleeding control, stabilize anticoagulation, escalate RV support when needed
Stable outpatient follow-up	Is the patient stable across flow, hemocompatibility, infection, and end-organ domains?	Symptoms, MAP/BP, INR and adherence, hemoglobin, LDH, renal/liver function, driveline score/site findings, alarms and device log, interval echo including AV opening and AR severity	Reinforce education, adjust BP/anticoagulation, investigate anemia/hemolysis, standardize driveline care, adjust follow-up intensity by risk
Deterioration/recurrent admission	Which complication is driving instability, and has the escalation threshold been crossed?	Focused echo or ramp study when stable, device log review, hemolysis panel, cultures and inflammatory markers, CT when cannula/outflow/deep infection suspected, neurologic assessment, renal/hepatic trend	Multidisciplinary review, intensify surveillance, treat driver complication, consider TAVR/SAVR, RV support, device exchange, transplant pathway, or palliative goals when appropriate

AR: aortic regurgitation; AV: aortic valve; BP: blood pressure; CT: computed tomography; FAC: fractional area change; INR: international normalized ratio; LDH: lactate dehydrogenase; LV: left ventricular; LVAD: left ventricular assist device; MAP: mean arterial pressure; PAP: pulmonary artery pressure; PAPi: pulmonary artery pulsatility index; PVR: pulmonary vascular resistance; RA/PCWP: right atrial/pulmonary capillary wedge pressure; RV: right ventricular; SAVR: surgical aortic valve replacement; TAPSE: tricuspid annular plane systolic excursion; TAVR: transcatheter aortic valve replacement; TEE: transesophageal echocardiography; TR: tricuspid regurgitation.

**Figure 1:**
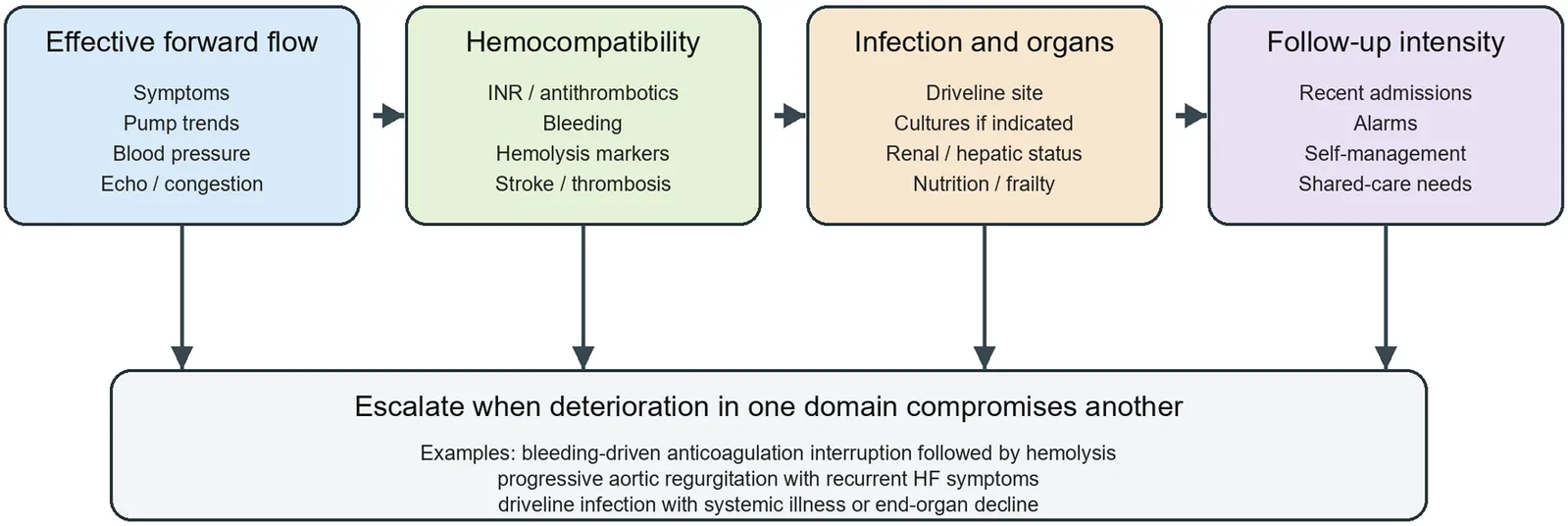
Four-domain surveillance and escalation framework for durable LVAD support. LVAD: left ventricular assist device.

## Overview of Major Complications After LVAD Implantation

Despite substantial improvement in outcomes after durable LVAD implantation, complications remain common and often determine whether support remains successful over the long term [2, 3, 6, 7]. For practical purposes, these complications can be grouped into hemodynamic complications, hemocompatibility-related adverse events, infectious complications, and end-organ dysfunction. Guideline- and consensus-based definitions are essential because similar symptoms may reflect different mechanisms. Post-LVAD RHF is generally defined by persistent or recurrent RV dysfunction requiring prolonged inotropes, inhaled or intravenous pulmonary vasodilators, right-sided mechanical circulatory support, or associated signs of venous congestion and end-organ dysfunction after LVAD implantation [4, 8]. Hemocompatibility-related adverse events include pump thrombosis, ischemic or hemorrhagic stroke, systemic embolism, hemolysis, and bleeding events requiring medical intervention, transfusion, hospitalization, or modification of antithrombotic therapy [2, 5–7, 9–14].

Hemodynamic complications mainly include RHF and progressive aortic regurgitation. These problems arise largely from altered ventricular interaction, RV preload and afterload imbalance, and chronic changes in valvular opening patterns during continuous-flow support [2, 4, 5, 8]. Hemocompatibility-related adverse events include pump thrombosis, systemic thromboembolism, hemolysis, and bleeding, particularly gastrointestinal bleeding. These events are closely related to non-physiological shear stress, platelet dysfunction, acquired von Willebrand syndrome, and the need for long-term antithrombotic therapy [2, 6, 7, 10, 11, 13].

Infectious complications, especially driveline infection and deeper device-related infection, remain a major source of late morbidity [15–18]. The 2024 International Society for Heart and Lung Transplantation (ISHLT) definitions distinguish mechanical circulatory support (MCS)-specific from non-MCS-specific infections [18]. Driveline infection should therefore be described by depth and extent rather than by erythema alone: superficial exit-site inflammation differs clinically from deep soft-tissue, tunnel, pump-pocket, or systemic infection. Renal dysfunction is also common during durable LVAD support and may reflect the combined effects of pre-existing heart failure, venous congestion, perioperative injury, and RV dysfunction [19]. Other end-organ complications and LVAD-related emergencies further complicate long-term care and often contribute to recurrent hospitalization [20].

Taken together, these complications reflect how device design, perioperative care, anticoagulation strategy, surveillance, and multidisciplinary management all shape long-term outcomes.

Routine follow-up can be organized around four recurring domains: (1) effective forward flow, assessed by symptoms, pump trends, blood pressure, echocardiography, and congestion; (2) hemocompatibility balance, assessed by anticoagulation stability, bleeding, hemolysis markers, and thromboembolic events; (3) infection and end-organ status, assessed by driveline findings, cultures when indicated, renal/hepatic function, nutrition, and frailty; and (4) follow-up intensity, adjusted according to recent admissions, alarms, recurrent adverse events, and patient self-management capacity. Escalation is appropriate when deterioration in one domain begins to compromise another, such as bleeding-driven anticoagulation interruption followed by hemolysis, progressive aortic regurgitation (AR) with recurrent heart failure symptoms, or driveline infection with systemic illness.

## RHF: Risk Stratification and Perioperative Management

### Pathophysiological mechanisms

RHF remains one of the defining and most difficult complications after LVAD implantation. RHF is multifactorial and may involve impaired RV-pulmonary vascular coupling under the new loading conditions created by left ventricular (LV) unloading. LVAD activation increases systemic flow and venous return, while leftward septal shift may impair RV geometry, tricuspid leaflet coaptation, and contractile efficiency. If pulmonary vascular resistance remains elevated, the RV faces simultaneous preload stress, afterload mismatch, septal dependence, and perioperative inflammatory or ischemic injury [4, 8, 21, 22].

Perioperative factors such as cardiopulmonary bypass, ischemia-reperfusion injury, volume shifts, bleeding, and inflammatory activation may further impair RV performance. Late RHF may also develop during chronic support and may be associated with persistent pulmonary vascular disease, progressive valvular dysfunction, arrhythmias, or suboptimal LVAD speed settings [4, 5, 8].

### Preoperative predictors and risk stratification

Accurate prediction of RHF after LVAD implantation remains difficult, but risk stratification should be structured rather than impressionistic. Preoperative assessment should integrate echocardiography, right heart catheterization, laboratory evidence of congestion, and clinical trajectory. Useful echocardiographic variables include RV size, qualitative RV systolic function, tricuspid regurgitation severity, tricuspid annular plane systolic excursion (TAPSE), tissue Doppler S', fractional area change, RV free-wall strain when available, septal position, and inferior vena cava congestion. Hemodynamic variables include right atrial pressure, pulmonary artery pressure, pulmonary vascular resistance, pulmonary capillary wedge pressure, cardiac index, right atrial pressure/pulmonary capillary wedge pressure ratio, pulmonary artery pulsatility index, and RV stroke work index [4, 8, 21, 22].

Several risk scores have been proposed, including the Michigan Right Ventricular Failure Risk Score, CRITT score, EUROMACS-RHF score, and other models incorporating renal dysfunction, hepatic dysfunction, vasopressor requirement, right atrial pressure, Interagency Registry for Mechanically Assisted Circulatory Support (INTERMACS) profile, and RV dysfunction. The EUROMACS-RHF score, for example, incorporates INTERMACS class, use of multiple inotropes, severe RV dysfunction on echocardiography, right atrial pressure/pulmonary capillary wedge pressure ratio, and hemoglobin, with reported early severe RHF risk ranging from approximately 11% in low-risk patients to more than 40% in high-risk patients [8]. These scores can support preoperative discussion and planning, but none should replace invasive hemodynamic assessment and multidisciplinary judgment because performance varies across devices, eras, and institutional practices [4, 8, 21].

### Prevention strategies

Prevention of RHF begins before surgery. Optimization of volume status means active decongestion until systemic venous congestion improves, including lower right atrial pressure, improved renal and hepatic indices, reduced peripheral edema or ascites, and acceptable filling pressures rather than simply achieving a lower body weight. Reduction of pulmonary vascular resistance may include oxygenation, correction of acidosis and hypercapnia, treatment of sleep-disordered breathing or pulmonary disease when relevant, cautious use of inotropes, and selective pulmonary vasodilators such as inhaled nitric oxide, inhaled prostacyclin, or phosphodiesterase-5 inhibition in appropriate patients [4, 8]. Careful patient selection and preoperative hemodynamic stabilization may reduce the likelihood of perioperative RV decompensation.

Intraoperatively, efforts should focus on minimizing RV ischemic injury, avoiding excessive transfusion, preventing hypoxia, hypercapnia, acidosis, and high airway pressures, and preventing abrupt septal displacement caused by aggressive pump speed settings. Transesophageal echocardiography should be used to assess RV size and function, septal position, tricuspid regurgitation, LV decompression, inflow cannula orientation, aortic valve opening, and adequacy of preload. Early postoperative surveillance should combine echocardiography, invasive hemodynamics when needed, renal and hepatic function, lactate, urine output, and serial congestion assessment [4, 8]. Prompt recognition of RV dysfunction may allow intervention before progression to refractory hemodynamic failure [4].

### Management

Management of RHF after LVAD implantation includes decongestion, inotropes, pulmonary vasodilators, rhythm control, ventilatory optimization, and careful pump-speed adjustment. Pump speed should be titrated to maintain effective LV unloading without excessive leftward septal shift, suction events, worsening tricuspid regurgitation, or collapse of LV dimensions. In stable patients, echocardiography-guided ramp testing can help evaluate LV size, septal position, aortic valve opening, mitral regurgitation, RV response, estimated filling pressures, and pump parameters across speed settings. In unstable perioperative patients, intraoperative or bedside echo-guided speed adjustment is often more practical than formal ramp testing. In selected patients with severe or refractory RHF, temporary RV mechanical support should be considered early rather than after irreversible renal, hepatic, or metabolic deterioration [4, 8, 21, 22].

In practice, RHF is easier to manage when approached as a perioperative syndrome that requires planned prevention, not rescue treatment alone.

## AR: Mechanisms, Surveillance, and Intervention

### Mechanisms

AR is recognized with increasing frequency during chronic LVAD support, particularly when continuous-flow support is prolonged. Persistent reduction in native transvalvular pulsatility may contribute to incomplete aortic valve opening, commissural fusion, leaflet remodeling, and progressive valvular incompetence. Altered aortic root flow patterns may also contribute to de novo or worsening AR [23, 24].

### Clinical impact and surveillance

Progressive AR may create a blind loop in which blood recirculates from the aorta into the LV and back through the pump, thereby reducing effective systemic output despite apparently acceptable device flow. Clinically, this may present with recurrent heart failure symptoms, reduced exercise tolerance, higher filling pressures, and repeated hospitalization [23, 24]. Echocardiography remains central to surveillance, but should be interpreted alongside pump parameters and overall clinical hemodynamics [23, 24].

### Management

Management depends on severity, symptoms, mechanism, and surgical candidacy. Mild AR may initially be managed conservatively through blood pressure control and pump optimization, with attention to maintaining intermittent aortic valve opening when feasible. More advanced disease may require surgical aortic valve repair, closure, replacement, redo surgical aortic valve replacement, or transcatheter aortic valve replacement in selected high-risk patients [23–25]. In LVAD-supported patients, TAVR can be technically challenging because of annular dilation, limited calcification, altered root flow, and device-related hemodynamics, but it may provide a less invasive option for severe symptomatic AR in patients at prohibitive surgical risk. Redo SAVR or valve closure may provide more definitive correction in selected patients but carries substantial operative risk. Aortic root thrombosis or aortic valve thrombus should also be considered when the aortic valve remains persistently closed, particularly in the setting of low pulsatility, inadequate anticoagulation, or embolic events [23–25]. In practice, the central question is whether AR has become hemodynamically significant enough to compromise effective support [23, 24, 26–29].

## Pump Thrombosis and Thromboembolic Events: Optimizing Antithrombotic Balance

### Pathophysiology and risk factors

Pump thrombosis and thromboembolic complications remain major concerns despite substantial improvements in device design and hemocompatibility with contemporary continuous-flow LVADs. Their development is multifactorial and reflects the interplay of altered flow dynamics, blood-material interaction, incomplete endothelial adaptation, patient-specific prothrombotic tendency, subtherapeutic anticoagulation, infection, and device-related factors such as inflow or outflow malposition [2, 9, 11–14]. In the HeartMate 3 era, suspected or confirmed pump thrombosis is uncommon compared with earlier axial-flow devices. In the 5-year MOMENTUM 3 follow-up, device thrombosis was uncommon with the fully magnetically levitated centrifugal pump, compared with a substantially higher rate in the axial-flow comparator group, while stroke and bleeding remained clinically important residual risks [3]. Registry reports similarly show that hemocompatibility-related adverse events have decreased but not disappeared [6, 7, 11].

Clinically, thrombosis in LVAD recipients rarely behaves like an isolated mechanical event. It often develops in the setting of broader hemocompatibility disturbance. Hemolysis, inflammatory activation, low-grade flow disturbance, or concurrent infection may all precede overt thrombosis. Likewise, strategies that reduce bleeding risk too aggressively may increase thrombotic vulnerability in selected patients.

### Clinical presentation and diagnostic evaluation

The presentation of pump thrombosis is often heterogeneous. Patients may develop progressive heart failure symptoms, low-flow alarms, increased pump power consumption, dark urine, laboratory evidence of hemolysis, or recurrent embolic events [2, 11, 12, 27]. Lactate dehydrogenase remains one of the most practical biomarkers in clinical monitoring, although interpretation should be integrated with plasma-free hemoglobin, pump parameters, symptoms, and imaging findings [12].

Echocardiography and device log review remain central to evaluation, while computed tomography (CT) may help define cannula position or outflow graft abnormalities. Because delayed recognition can lead to refractory hemolysis, stroke, or urgent reoperation, diagnostic escalation should not be postponed when clinical suspicion is high [20, 27].

### Prevention and treatment strategies

The prevention of pump thrombosis depends largely on maintaining antithrombotic balance. During implantation, unfractionated heparin is commonly administered before cardiopulmonary bypass with an activated clotting time (ACT) target determined by institutional cardiopulmonary bypass protocols. After surgery, intravenous heparin is usually restarted only after surgical bleeding is controlled, chest tube output is acceptable, and coagulation status is stable; many centers begin low-dose heparin within the first 12–24 h and transition toward therapeutic anticoagulation over the following days, but timing must be individualized [5, 9]. Long-term therapy has traditionally used warfarin with a device-specific international normalized ratio (INR) target and aspirin, although the need for aspirin is being reconsidered in HeartMate 3 recipients after the ARIES-HM3 trial showed that aspirin avoidance reduced nonsurgical bleeding without increasing thrombotic events in appropriately selected patients [30]. Direct oral anticoagulants should not be used routinely for durable LVAD anticoagulation outside clinical trials or highly selected specialist-directed circumstances. Prior experience with dabigatran in mechanical circulatory support raised safety concerns, and evidence for factor Xa inhibitors remains insufficient for broad recommendation. Therefore, vitamin K antagonist-based anticoagulation remains the standard backbone for most durable LVAD recipients, with antiplatelet therapy individualized by device type, bleeding history, thrombotic risk, and institutional protocol [5, 30].

When pump thrombosis is suspected or confirmed, treatment options include intensification of anticoagulation, short-term parenteral therapy, thrombolysis in carefully selected patients, or surgical pump exchange when conservative treatment is ineffective or unsafe [5, 20]. The choice depends on hemodynamic status, neurological risk, bleeding risk, and the severity of device dysfunction.

### Stroke and systemic thromboembolism

Stroke remains one of the most serious complications during LVAD support because of its major impact on mortality, disability, and subsequent treatment options. Risk is shaped by antithrombotic control, device-related thrombogenicity, blood pressure management, infection, and patient comorbidity [2, 11, 14, 31]. Contemporary registry and trial data suggest that stroke remains a clinically meaningful event even with magnetically levitated devices; stroke remains a concern during long-term HeartMate 3 support, although rates vary according to device era, follow-up duration, event definitions, blood pressure control, infection burden, and antithrombotic strategy [2, 3, 6, 7, 14, 31]. Prevention therefore requires integrated management, including stable anticoagulation, infection control, blood pressure optimization, and early recognition of hemolysis or device dysfunction [5, 14, 31].

## Bleeding Complications: Hemocompatibility-Related Injury and Clinical Management

### Mechanisms of bleeding in continuous-flow LVAD support

Bleeding is among the most frequent complications following LVAD implantation and remains a major cause of recurrent hospitalization [2, 6, 13]. Its pathophysiology extends beyond simple over-anticoagulation. Continuous-flow devices expose blood components to non-physiological shear stress, contributing to platelet dysfunction, degradation of high-molecular-weight von Willebrand factor multimers, and acquired von Willebrand syndrome. Prior mechanistic and clinical discussions suggest that von Willebrand factor degradation is an important link between shear stress, hemolysis, and bleeding during LVAD support. Reduced pulsatility may also promote gastrointestinal angiodysplasia, particularly during long-term device support [13].

The clinical result is a fragile hemostatic balance in which even otherwise standard antithrombotic regimens may provoke recurrent bleeding. This explains why bleeding remains common even in patients with apparently therapeutic anticoagulation levels and why management often requires repeated reassessment rather than one-time correction.

### Gastrointestinal bleeding

Gastrointestinal bleeding is the most characteristic bleeding phenotype during durable LVAD support. It may present as overt upper or lower gastrointestinal hemorrhage, recurrent occult blood loss with anemia, or repeated hospital admissions for transfusion and endoscopic evaluation. The recurrence rate is clinically significant and often contributes to treatment fatigue, impaired quality of life, and repeated interruption of anticoagulation [6, 7, 13].

The pathogenesis is multifactorial, involving mucosal hypoperfusion, vascular malformation, altered angiogenic signaling, acquired von Willebrand syndrome, and chronic anticoagulant exposure. Because of this complexity, endoscopic management alone may not fully prevent recurrence [13].

### Management principles

Management of bleeding should begin with hemodynamic stabilization and risk stratification. Temporary adjustment of anticoagulation or antiplatelet therapy may be required, but this must be balanced carefully against the competing risk of pump thrombosis or stroke [5, 10, 11, 13, 14]. Endoscopic evaluation is appropriate for gastrointestinal bleeding when clinically indicated, and transfusion strategies should be individualized according to hemodynamic status and comorbidity.

In patients with recurrent bleeding, management often shifts from treatment of isolated episodes to long-term recurrence prevention. This may include reassessment of antithrombotic intensity, optimization of blood pressure, evaluation of device parameters, and in selected cases a broader reassessment of overall support strategy [5–7, 13, 30].

### Prevention strategies

Prevention of bleeding in LVAD recipients requires more than conservative anticoagulation alone. It also depends on minimizing unnecessary antithrombotic exposure, monitoring for recurrent anemia or occult bleeding, and recognizing patients in whom a lower-intensity strategy may be reasonable. In the contemporary era, there is growing interest in individualized regimens, particularly in HeartMate 3 recipients, but careful monitoring remains essential [5, 10, 13, 30].

## Infection and Driveline-Related Complications

### Clinical spectrum

Infectious complications remain a persistent limitation of long-term LVAD support. Driveline infections are the most common device-related infections, although deeper pump or pocket infections and bloodstream infections also occur. These events can lead to recurrent hospitalization, compromise transplant candidacy, and adversely affect long-term survival [5, 18, 32].

### Risk factors and diagnosis

Multiple factors contribute to infection risk, including diabetes, malnutrition, renal dysfunction, prolonged support duration, inadequate exit-site care, local trauma, and repeated dressing disruption. Diagnosis requires careful attention to the depth and extent of infection. Superficial exit-site inflammation must be distinguished from deeper tissue involvement, tunnel infection, or systemic infection, as management strategies differ substantially.

Clinical examination, wound assessment, microbiological cultures, inflammatory markers, and selected imaging studies all contribute to diagnosis. Recommended surveillance should include exit-site pain, erythema diameter, warmth, swelling, induration, drainage amount and character, granulation tissue, driveline trauma or instability, fever, leukocytosis, C-reactive protein when clinically indicated, blood cultures for systemic illness, and ultrasound or CT/positron emission tomography (PET)-CT when deep extension is suspected [15–18]. Recurrent or indolent symptoms should be taken seriously, particularly in patients on chronic support.

### Treatment and prevention

Treatment generally includes targeted antimicrobial therapy, local wound care, and, in some cases, surgical intervention. In selected patients who are not immediate candidates for device exchange or transplantation, chronic suppressive treatment may also be necessary. Prevention is the more important long-term strategy and depends heavily on patient education, driveline stabilization, standardized dressing protocols, and regular outpatient surveillance [5, 18, 32, 33]. Recent quality-improvement work has emphasized structured infection-reduction toolkits and standardized preventive workflows across centers, suggesting that infection prevention should be built into routine care rather than left to local improvisation [33].

Recent prospective work has explored additional preventive approaches, including local prophylactic strategies and alternative driveline positioning techniques, but the evidence remains limited [16, 17].

Further progress may come from fully implantable or wirelessly powered systems that reduce long-term reliance on percutaneous hardware.

## Renal Dysfunction and Other End-Organ Complications

Renal dysfunction is common both before and after LVAD implantation and reflects the combined effects of advanced heart failure, venous congestion, low cardiac output, perioperative injury, and RV dysfunction [19]. Some patients improve after LV unloading, whereas others develop persistent or progressive renal impairment, particularly when postoperative congestion, RHF, or repeated complications persist [5, 19].

Renal function has direct implications for anticoagulation safety, infection risk, diuretic responsiveness, and transplant candidacy. For that reason, it deserves longitudinal follow-up alongside hemodynamic assessment rather than being treated as a secondary comorbidity.

Other end-organ complications, including hepatic dysfunction, malnutrition, frailty progression, and arrhythmias, may likewise reflect the overall adequacy of support and the cumulative burden of adverse events [5, 19, 20]. These problems often interact and contribute to recurrent hospitalization and impaired functional recovery.

## Integrated Follow-Up and Multidisciplinary Prevention Strategies

Modern LVAD care requires a structured multidisciplinary framework that extends far beyond the index hospitalization [1, 4, 5]. Surgeons, advanced heart failure cardiologists, anticoagulation teams, imaging specialists, infectious disease physicians, rehabilitation staff, and specialized nurses all contribute to long-term success. LVAD-related complications rarely occur in isolation; they tend to recur and often interact with one another.

Routine follow-up should include symptom review, blood pressure assessment, anticoagulation monitoring, surveillance for hemolysis and infection, renal function evaluation, device parameter review, and interval echocardiography when clinically indicated [1, 4, 5, 18]. Standardized follow-up pathways may help detect evolving problems before they lead to major hemodynamic or infectious deterioration. Clinical consensus recommendations support interdisciplinary follow-up for surveillance, patient education, and coordinated management of recurrent complications [5]. Day-to-day pharmacological optimization is an integral part of durable outpatient care [5, 34]. In both intensive care unit (ICU) and outpatient settings, familiarity with common LVAD-related emergencies remains increasingly important as the supported population expands [5, 20].

The Heart Failure Society of America (HFSA) expert consensus on durable mechanical circulatory support also underscores the importance of longitudinal pharmacologic optimization, blood pressure control, anticoagulation stewardship, and structured treatment pathways for recurrent LVAD-related complications [5].

For daily practice, a useful surveillance framework can be organized around four recurring questions: Is effective forward flow being maintained? Is hemocompatibility still balanced? Is there evidence of infection or end-organ decline? Has the current follow-up intensity matched the patient’s present risk? Framing outpatient care in this way may help teams move from reactive complication treatment toward earlier prevention-oriented intervention.

Patient education is equally important. Durable support depends in part on whether patients can recognize driveline problems, respond appropriately to device alarms, follow anticoagulation and dressing instructions, and seek timely care for bleeding, infection, or worsening heart failure symptoms. In that sense, multidisciplinary care is not merely an organizational framework; it is part of treatment itself.

## Future Directions

Further improvement in LVAD outcomes will likely depend on both technological advances and better long-term management [2, 3, 5]. Priority areas include improved hemocompatibility, more individualized antithrombotic strategies, better prediction of RV failure and recurrent bleeding, and continued refinement of surgical and postoperative care pathways [2–5, 11].

Important directions include fully implantable systems and wireless energy-transfer technology to reduce driveline-related infection, together with data-driven surveillance and biomarker-based risk stratification to support earlier detection of complications and more individualized intervention.

The goal is not only longer survival, but safer support over time, better functional recovery, and more acceptable quality of life.

## Conclusions

Durable LVAD therapy has reshaped the management of advanced heart failure, but major complications remain the principal barrier to sustained long-term benefit [1–4]. RHF, aortic regurgitation, pump thrombosis, bleeding, infection, and end-organ dysfunction are closely linked to altered hemodynamics, continuous-flow physiology, hemocompatibility-related injury, and the practical complexity of prolonged device support. Better outcomes depend not only on successful implantation, but also on careful longitudinal surveillance, earlier recognition of evolving complications, and coordinated multidisciplinary management.

The practical value of a contemporary LVAD review lies less in repeating that these complications exist than in clarifying where prevention can be strengthened, how surveillance should be structured, and when management must escalate across the perioperative and chronic phases of support. A complication-centered, management-oriented approach may therefore offer greater clinical utility than a purely descriptive summary of adverse events.

Good outcomes depend on more than technical success in the operating room. They also require appropriate patient selection, perioperative RV protection, careful antithrombotic management, close surveillance, timely intervention, and coordinated multidisciplinary follow-up. Continued advances in both device technology and longitudinal care pathways should help reduce adverse events and improve the durability of LVAD support.

## Data Availability

Not applicable. This article is a narrative review and does not report original datasets generated or analyzed by the authors.

## Abbreviations

AI: artificial intelligence
AR: aortic regurgitation
CT: computed tomography
GI: gastrointestinal
HFSA: Heart Failure Society of America
INTERMACS: Interagency Registry for Mechanically Assisted Circulatory Support
LVAD: left ventricular assist device
MDT: multidisciplinary team
RHF: right heart failure
RV: right ventricular
